# Genetic Epilepsies and Developmental Epileptic Encephalopathies with Early Onset: A Multicenter Study

**DOI:** 10.3390/ijms25021248

**Published:** 2024-01-19

**Authors:** Benedetta Cavirani, Carlotta Spagnoli, Stefano Giuseppe Caraffi, Anna Cavalli, Carlo Alberto Cesaroni, Gianni Cutillo, Valentina De Giorgis, Daniele Frattini, Giulia Bruna Marchetti, Silvia Masnada, Angela Peron, Susanna Rizzi, Costanza Varesio, Luigina Spaccini, Aglaia Vignoli, Maria Paola Canevini, Pierangelo Veggiotti, Livia Garavelli, Carlo Fusco

**Affiliations:** 1 Child Neuropsychiatry Unit, Azienda USL di Parma, 43121 Parma, Italy; benedetta.cavirani@gmail.com; 2 Child Neurology and Psychiatry Unit, Department of Pediatrics, Presidio Ospedaliero Santa Maria Nuova, AUSL-IRCCS di Reggio Emilia, 42122 Reggio Emilia, Italy; anna.cavalli@ausl.re.it (A.C.); carloalberto.cesaroni@ausl.re.it (C.A.C.); daniele.frattini@ausl.re.it (D.F.); susanna.rizzi@ausl.re.it (S.R.); carlo.fusco@ausl.re.it (C.F.); 3 Medical Genetics Unit, Presidio Ospedaliero Santa Maria Nuova, AUSL-IRCCS di Reggio Emilia, 42122 Reggio Emilia, Italylivia.garavelli@ausl.re.it (L.G.); 4 Pediatric Neurology Unit, Department of Pediatric Neurology, Buzzi Children’s Hospital, 20154 Milan, Italy; gianni.cutillo@gmail.com (G.C.); silvia.masnada1986@gmail.com (S.M.); pierangelo.veggiotti@unimi.it (P.V.); 5 Department of Brain and Behavioural Sciences, University of Pavia, 27100 Pavia, Italy; valentina.degiorgis@unipv.it (V.D.G.); costanza.varesio@mondino.it (C.V.); 6 Department of Child Neurology and Psychiatriy, IRCCS Mondino Foundation, ERN-Epicare, 27100 Pavia, Italy; 7 Medical Genetics Unit, Woman-Child-Newborn Department, Fondazione IRCCS Ca’ Granda, Ospedale Maggiore Policlinico, 20122 Milan, Italy; giuliabruna.marchetti@unimi.it; 8 Medical Genetics, Meyer Children’s Hospital IRCCS, 50139 Florence, Italy; angela.peron@unifi.it; 9 Department of Experimental and Clinical Biomedical Sciences “Mario Serio”, Università degli Studi di Firenze, 50121 Florence, Italy; 10 Medical Genetics, ASST Santi Paolo e Carlo, San Paolo Hospital, 20142 Milan, Italy; 11 Clinical Genetics Unit, Department of Obstetrics and Gynecology, V. Buzzi Children’s Hospital, University of Milan, 20157 Milan, Italy; luigina.spaccini@asst-fbf-sacco.it; 12 Child Neuropsychiatry Unit-Epilepsy Center, ASST Santi Paolo e Carlo, San Paolo Hospital, 20142 Milan, Italy; aglaia.vignoli@unimi.it (A.V.); mariapaola.canevini@unimi.it (M.P.C.); 13 Department of Health Sciences, University of Milan, 20157 Milan, Italy; 14 Department of Biomedical and Clinical Sciences, University of Milan, 20157 Milan, Italy

**Keywords:** epilepsy, developmental and epileptic encephalopathies, outcome, genetics, drugresistance, behaviour, intellectual disability, developmental delay, neurodevelopmental disorders

## Abstract

The genetic causes of epilepsies and developmental and epileptic encephalopathies (DEE) with onset in early childhood are increasingly recognized. Their outcomes vary from benign to severe disability. In this paper, we wished to retrospectively review the clinical, genetic, EEG, neuroimaging, and outcome data of patients experiencing the onset of epilepsy in the first three years of life, diagnosed and followed up in four Italian epilepsy centres (Epilepsy Centre of San Paolo University Hospital in Milan, Child Neurology and Psychiatry Unit of AUSL-IRCCS di Reggio Emilia, Pediatric Neurology Unit of Vittore Buzzi Children’s Hospital, Milan, and Child Neurology and Psychiatry Unit, IRCCS Mondino Foundation, Pavia). We included 168 patients (104 with monogenic conditions, 45 with copy number variations (CNVs) or chromosomal abnormalities, and 19 with variants of unknown significance), who had been followed up for a mean of 14.75 years. We found a high occurrence of generalized seizures at onset, drug resistance, abnormal neurological examination, global developmental delay and intellectual disability, and behavioural and psychiatric comorbidities. We also documented differing presentations between monogenic issues versus CNVs and chromosomal conditions, as well as atypical/rare phenotypes. Genetic early-childhood-onset epilepsies and DEE show a very wide phenotypic and genotypic spectrum, with a high risk of complex neurological and neuropsychiatric phenotypes.

## 1. Introduction

Epilepsy is the most frequently occurring neurological disease in the pediatric age range. Onset in early childhood is common. In 2017, the International League Against Epilepsy (ILAE) proposed a revision of previous classifications for seizures and epilepsy. This latest classification incorporates six possible aetiologic categories into a taxonomy: genetic, structural (which may be congenital or acquired), metabolic, immune, infectious, and unknown [1].

Genetic epilepsies are diseases arising from a known or presumed causative genetic variation, resulting in seizures as the principal clinical hallmark [1]. Genetic/presumed genetic aetiologies are estimated to represent approximately 20–30% of all epilepsies [2,3,4,5].

On a clinical basis, genetic epilepsies can be distinguished into generalized genetic epilepsies, focal genetic epilepsies, and developmental and epileptic encephalopathies (DEE) [1]. 

Technological developments, in particular with the availability of next-generation sequencing (NGS) techniques, have brought exome (ES) and genome (GS) sequencing into clinics, allowing the identification of a growing number of genes linked to epilepsy. Nowadays, more than 500 epilepsy-linked genes have been identified [6]. 

However, several cases show variants of uncertain significance (VUS), and it is not always possible to identify (likely) pathogenic variants [7,8,9], leaving some cases unsolved [10,11]. 

The aim of this multicentre study was to analyze a cohort of patients with genetic epilepsies or DEE with onset in the first three years of life. In particular, the primary objective of this study was to report on a detailed phenotypic description, starting from genotypes, including clinical, EEG, neuroimaging, and seizure outcome data. 

## 2. Results

In total, we enrolled 168 patients (97 females and 71 males) with a range of genetic epilepsies and or developmental and epileptic encephalopathies with onset within the first 3 years of age. 

Mean age at epilepsy onset was 11 months (range 0–36 months) and mean age at last follow-up was 177 months (range 0–672 months).

Among the 168 patients, 149 (88.6%) carried a pathogenic or likely pathogenic variant and 19 (11.4%) carried a VUS. Among patients with pathogenic (P) or likely pathogenic (LP) variants, we distinguished between monogenic conditions (104 patients) and chromosomal abnormalities (45 patients). The 19 patients with VUS were described separately. Some of these patients’ descriptions have already been published [12,13,14,15,16,17,18,19,20,21,22,23].

### 2.1. Monogenic Conditions

**Figure 1:**
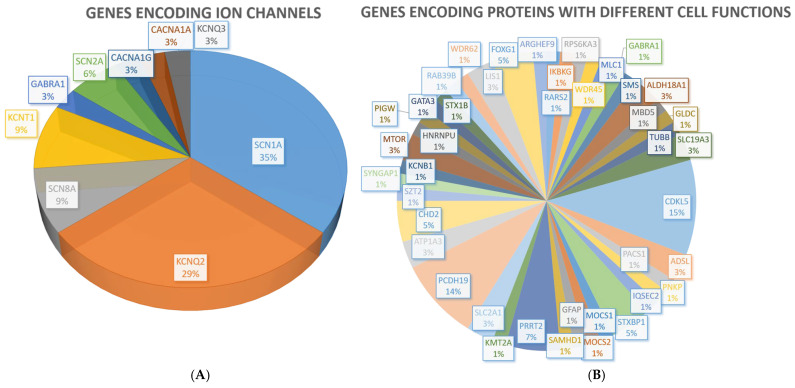
(**A**) Distribution of aetiological diagnoses within genes encoding ion channels in our cohort. (**B**) Distribution of aetiological diagnoses involving other cell functions.

In this group, 59 patients carried P variants, while 45 had an LP variant. The single largest functional group of single-gene variants (36 patients; 34.6%) was represented by genes encoding ion channels (Table 1 and Figure 1): *SCN1A* (11 patients), *KNCQ2* (9 patients), *SCN8A* (4 patients), *KCNT1* (3 patients), *CACNA1G* (1 patient), and *SCN2A* (2 patients). 

Several variants were reported in additional genes encoding proteins with different cell functions (Table 1), of which 28 were identified in single patients (Figure 1).

#### 2.1.1. Clinical Findings

##### Family History 

In most patients (63/104, 60%), family history was negative for neurological diseases. The remaining patients (41/104, 40%) had a positive family history for neurological or neurodevelopmental disorders. 

##### Epilepsy

The main seizure type at onset was generalized (39/104, 37.5%). Among patients with generalized seizures at onset, 20 had tonic–clonic seizures, 12 had epileptic spasms (ES), 2 had myoclonic and myoclonic–atonic seizures (*CHD2*, *RAB39B*), 2 had tonic seizures (*ATP1A3*, *PIGW*), 1 had absence seizures (one typical and one atypical), and one had atonic seizures (*RPS6KA3*). The second group (32/104, 31%) presented focal seizures at onset (31 with motor seizures and one with a non-motor seizure). Finally, 15/104 (14%) started with febrile seizures and 13/104 (12.5%) had focal to bilateral tonic–clonic seizures. Three (3%) patients had status epilepticus at onset and two (2%) sisters, harbouring a compound heterozygous variant in the *ALDH18A1* gene, had developmental/epileptic encephalopathy with spike wave activation in sleep (DEE-SWAS) at onset. Within this subgroup of children with monogenic conditions, 48 (46%) had epilepsy and 56 (54%) DEE.

##### Electroencephalogram (EEG) Pattern at Onset

EEG at onset revealed the poor organization of background activities with or without interictal epileptiform discharge in 10 patients (10%), excess slow activity in 8 patients (8%), hypsarrhythmia in 4 patients (4%), and burst-suppression patterns in 3 patients (3%). Multifocal discharges were present in 16 patients (15%), focal discharges in 15 patients (14%), and generalized discharges in 11 patients (11%). DEE-SWAS was present in two patients (2%). One patient (1%) presented a Lennox–Gastaut pattern. EEG was normal in 16 patients (15%). For the remaining 18 patients (17%), data on EEG findings at onset were not available. 

##### Neurological Examination

In most patients (74/104, 71%), neurological examination was abnormal. The most frequent neurological signs were abnormal muscle tone (i.e., hypotonia and/or spasticity) and gait abnormalities (i.e., ataxia). Furthermore, stereotypic hand movements represented the most common movement disorder (associated with *CDKL5*, *FOXG1*, *KCNT1*, *KCNQ2*, *STZ2*, and *CACNA1G* variants). Extra-pyramidal signs (i.e., bradykinesia) were reported in only one patient with Dravet syndrome. Moreover, one patient with an *SCN1A* pathogenic variant presented with a complex neurological phenotype characterized by early-onset epileptic encephalopathy, severe developmental delay, and a hyperkinetic movement disorder (already described in [12]). Detailed characteristics (when available) are depicted in Table 2.

##### Neurodevelopmental Features and Psychiatric Comorbidities

Most patients (80.7%, 84/104) presented developmental delay (DD) and/or intellectual disability (ID). Only five patients presented speech delay and the remaining patients (15/104, 14.4%) had normal neurodevelopment. 

**Figure 2:**
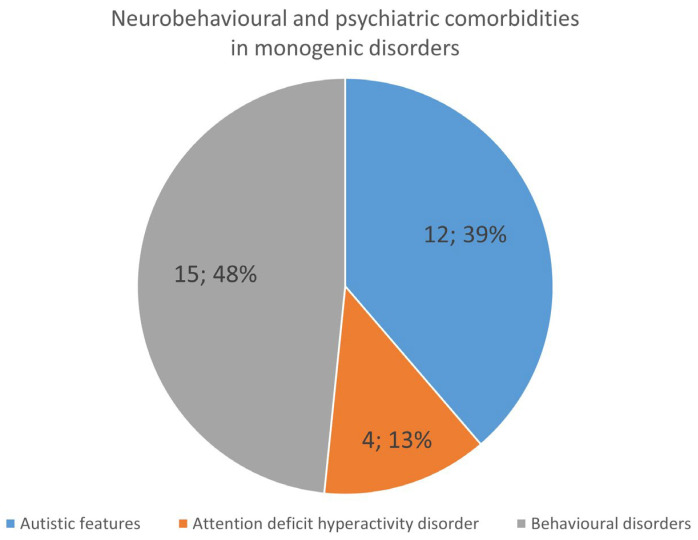
Behavioural and neuropsychaitric comorbidities in patients with monogenic disorders.

Autistic features were present in 12/104 (11.5%) patients (harbouring *KCNQ2*, *CACNA1G*, *ADSL*, *PNKP*, *STXBP1*, *MTOR*, *SCN1A*, *HNRNPU* and *PCDH19* variants). Attention deficit hyperactivity disorder (ADHD) was described in 4/104 (*PCDH19*, *CHD2* and *MBD5*). Other behavioral disorders were reported in 15/104 (*GLDC*, *PCAS1*, *MOCS1*, *GATA3*, *STX1B*, *PCDH19*, *SYNGAP1*, *GABRG2*, *SCN1A*, and *RAB39B*) (Figure 2 and Table 3).

##### Neuroimaging Findings

Brain MRI was unremarkable in nearly half of the patients (51/104, 49%), while 48 patients (46%) showed abnormalities findings during brain MRI (32 brain malformations, 16 progressive changes). Five patients had a CT scan. Neuroimaging data were not available in four patients. A detailed description of neuroimaging findings is reported in Table 4.

##### Genetic Testing 

In more than half of the patients (65/104, 62%), diagnosis was achieved using NGS techniques (i.e., single-gene sequencing, gene panel, and exome). For 17/104 (16.3%), Sanger sequencing identified the diagnostic variant, while MLPA did so for one (1%) patient (*IKBKG* gene). For five (4.8%) patients, Array-CGH was diagnostic (9q34.11 deletion including the *STXBP1* gene [13], 20q13.33 deletion including *KCNQ2* in two sisters, 16p11.2 deletion including *PRRT2*, and 14q12 deletion including *FOXG1*). For the remaining 16 (16/104, 15.4%) patients, although the causative/likely causative variant was known from the clinical charts, it was not possible to identify what specific diagnostic test was performed because the full report was unavailable for direct review. 

##### Segregation Analysis

Segregation analysis was performed and available for review in 80 patients (80/104, 76.9%). The detected variants occurred de novo in 54 patients (54/104, 51.9%). They were inherited from the patient’s mother in five cases (5/104, 4.8%) and from the patient’s father in seven cases (7/104, 6.7%). In 14 cases (14/104, 13.5%), the patient’s parents were heterozygous carriers. Information on segregation was not available in 24 cases (24/104, 23.1%). 

##### Seizure Outcome at the Latest Follow-Up Visit

At the latest follow-up visit, approximately half (54/104, 52%) of the patients had drug-resistant seizures. For two patients, data on outcomes were not available, while other patients (48/104, 46%) were seizure-free. Among seizure-free patients, 8/48 (16.7%) were not on regular antiseizure medications. 

##### EEG Pattern at the Latest Follow-Up Visit

EEG at latest follow-up visit revealed the poor organization of background activities, with frequent multifocal or generalized discharges in 32 patients (31%), focal epileptiform discharges in 12 patients (12%), multifocal discharges in 7 patients (7%), and diffuse discharges in 7 patients (8%). Excess slow activity with or without interictal epileptiform discharges was seen in 11 cases (11%) and there was a DEE-SWAS pattern in three patients (3%). One patient (1%) presented a Lennox–Gastaut pattern. In 25 patients (24%), EEG was normal. For the remaining five patients (5%), data on EEG at the latest follow-up visit were not available for review. 

By comparing EEG features at onset and at the latest follow-up, we documented an improvement in 14 patients (13.5%) and a worsening in 10 (9.6%), while EEG findings were stable in 61 patients (58.6%). We were unable to comment on the evolution of EEG patterns in 19 cases (18.3%) because either the first or the last EEG (or both) were unavailable for review.

### 2.2. Chromosomal Abnormalities

**Figure 3:**
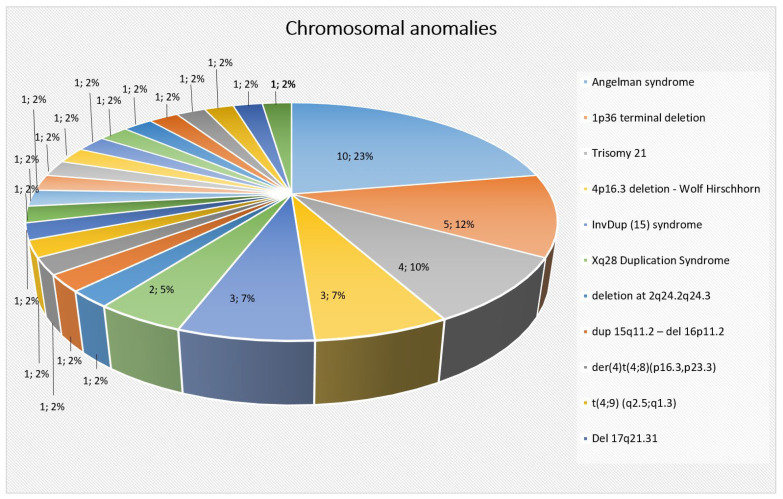
Distribution of chromosomal abnormalities and CNVs in our cohort.

In our cohort, the most frequent chromosomal abnormalities were the deletion of chromosome 15 at 15q11-q13—Angelman syndrome (10/45), 1p36 terminal deletion (5/45), trisomy 21 (4/45), 4p16.3 deletion resulting in Wolf–Hirschhorn (3/45), InvDup(15) syndrome (3/45), and Xq28 duplication syndrome (2/45). Additional chromosomal abnormalities (18 patients), each represented by a single patient, include: 46,XX,del(11)(q23.3q25), 46,XY,del(2)(q24.2q24.3), 46,XX,dup(15q11.2) 46,XX,del(16p11.2), del(4)t(4;8) (p16.3,p23.3),t(4;9)(q2.5;q1.3), 46,XY,del(17q21.31), 47,XX+13, 46,XY,del(6)(q26-qter), 16p11.2 microdeletion syndrome, 46,XX,del(16)(p13.11p12.3) pat, 46,XX,del(1q44), 46,XX,dup(14)(q11.2q12), 46,XX,del(5)(q11.2q13.2) and 46,XX,dup(5q13.2), 46,XX,del(8)(p23.3p23.2) and 46,Xxdup(13)(q32.1q34), 46,XX,del(6)(q21q22.31), 46,XX,del(9)(q33.3q34.11), 46,XX,del(9qter), 46,XY,del(17p13.3), and 46,XX,del(11)(q23.3q25) (Figure 3). 

#### 2.2.1. Clinical Findings

##### Family History 

In the majority of patients (32/45, 71%), family history was negative for neurological diseases. A small number of patients (12/45, 26%) had positive family history for neurological diseases (i.e., epilepsy, febrile seizures) or neurodevelopmental disorders (autism spectrum disorder, intellectual disability, and speech delay). Family history was not available for one adopted child.

##### Epilepsy

The main seizure type at onset is generalized (28/45, 62%). Among patients with generalized seizures at onset, 11 had epileptic spasms (ES), 7 tonic–clonic seizures, 3 myoclonic seizures, 2 atonic seizures, and 5 atypical absences (patients with Angelman syndrome). 

Six patients had focal-to-bilateral tonic–clonic seizures at onset (13%), while four (8.9%) had focal onset seizures (two motor and two non-motor). Five (5/45, 11.1%) patients presented with febrile seizures at onset and one (1/45, 2.2%) had status epilepticus at onset (2q24.2q24.3 deletion). Within this subgroup of children with chromosomal abnormalities and CNVs, 28 (62%) had epilepsy and 17 (38%) had DEE. 

##### Electroencephalogram Pattern (EEG) at Onset

EEG at onset documented abnormal background activity with multifocal or generalized discharges in seven patients (15%), focal or multifocal discharges in six patients (13%), hypsarrhythmia in four patients (9%), generalized discharges in two patients (4%), slow background activity in two patients (4%), and slow background activity with interictal epileptiform discharges in two patients (4%). One patient presented with a Lennox–Gastaut pattern (2%). In four patients, EEG was normal (9%). For the remaining 17 patients (37%), data on EEG at onset were not available. 

##### Neurological Examination

In most patients within this group (39/45, 87%), neurological examination results were abnormal. Gait abnormalities and abnormal muscle tone were the most common neurological signs. Detailed characteristics (when available) are depicted in Table 5.

##### Neurodevelopmental Features and Psychiatric Comorbidities

**Figure 4:**
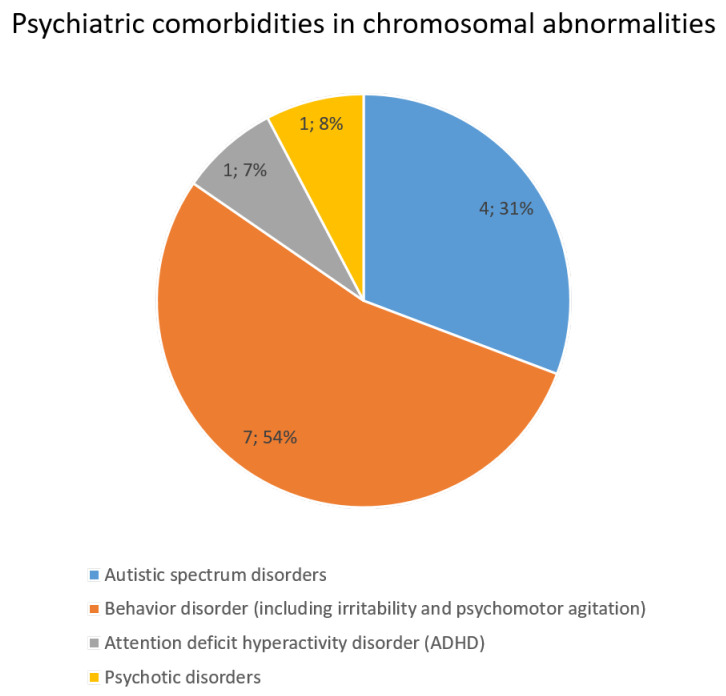
Behavioural and neuropsychiatric comorbidities in patients with chromosomal abnormalities and CNVs.

DD and/or ID are present in most patients within this group (91.1%, 41/45). Autistic features are described in 4/45 (1p36 microdeletion syndrome, 46,XX,del(1q44), Xq28 duplication syndrome), ADHD is present in one patient (46,XX,dup(16)(p13.11p12.3), while behavioral disorders (i.e., agitation/irritability, psychosis) are present in 15/45 [Angelman syndrome, Down’s syndrome, 1p36 microdeletion syndrome, 46,XX,dup(11)(q23.3q25), InvDup(15), Wolf–Hirschorn syndrome, 46,XX,del(6)(q21q22.31), and 46,XX,del(9qter)] (Figure 4). 

##### Neuroimaging Findings

More than half of the patients in this group had brain MRI abnormalities (25/45, 55%, with 21 showing brain malformation and four progressive changes). In 13/45 (29%) patients, brain MRI was unremarkable, while in 7 (16%) patients’ neuroimaging data were not available. A detailed description of neuroimaging findings is reported in Table 6.

##### Genetic Testing 

For most patients (30/45, 67%), the diagnosis was obtained using Array-CGH, while for 10/45 (22%) this was performed using karyotype. 

NGS gene panel led to a diagnosis for three patients [one patient 46,XX,del(4p16.3), and two patients with Angelman syndrome, the first carrying the c. 1347_1348delGA (p.Asn450Glnfs*23) variation on the *UBE3A* gene and the second a 46,XX,del(15)(q11q13)], while direct gene sequencing (*UBE3A* gene) was diagnostic for two patients.

##### Segregation Analysis

The detected CNVs occurred de novo in 20 cases (20/45, 44.4%), while in one case an Xq28 duplication syndrome was inherited from the proband’s mother (1/45, 2.2%). In 24 cases (24/45, 53.3%), this information was not available.

##### Seizure Outcome 

At the latest follow-up visit, more than half of the patients (26/45, 58%) were seizure-free and among these only two [one with Down’s syndrome and one with 46,XY,del(16p11.2)] were not on medication. The remaining patients (19/45, 42%) were drug-resistant.

##### EEG Pattern at the Latest Follow-Up Visit

EEG at the end of follow-up revealed the poor organization of background activity, with frequent multifocal or generalized discharges in 20 patients (44%), diffuse interictal epileptiform discharges in seven patients (16%), focal or multifocal interictal epileptiform discharges in five patients (11%), and an excess of slow activities with or without interictal discharges in three cases (7%). In seven patients (15%), EEG was normal. 

For the remaining three patients (7%), data on EEG at latest follow-up visit were not available. 

By comparing EEG features at onset and at the latest follow-up, we documented an improvement in five patients (11.1%) and a worsening in four (8.9%), while EEG findings were stable in 18 patients (40%). We are unable to comment on the evolution of EEG patterns in 18 cases (40%) because either the first or the last EEG (or both) were unavailable for review.

### 2.3. Genetic Variations of Unknown Clinical Significance

Nineteen patients in our cohort carried at least one VUS. Of these, five (5/19, 26.3%) had a CNV [46,XX,del(16p13.3); 46,XY,del(16p13.2); 46,XY,del(22q11.21); 46,Xxdel(16p13.11); 46,XY,dup(2p21) 46,XY,dup(16p13.3)] and 14/19 (73.7%) had a single-gene variant (*CLCN*; *SCN8A*; *m TOR*; *SIK1*; *WDR45*; *GRIN2A*; *KCNMA1*; *HUWE1*; *SCN1A and HDAC4*; *HCN1*; *DOCK3*; *SCN2A*; *SCN1A*) of unclear clinical significance. Nine (47.4%) were inherited from one parent (the mother in six cases and the father in three cases). Among involved single genes, 11 (57.9%) cause autosomal dominant disorders, 2 (10.5%) X-linked disorders, and 2 (10.5%) autosomal recessive disorders (Table 7).

#### 2.3.1. Clinical Findings

##### Family History

The majority of patients (11/19, 57.9%) had no family history of neurological diseases. However, more than one-third (7/19, 36.8%) had a positive family history. Family history was not available in one.

##### Epilepsy

The mean age at epilepsy onset was 17.1 months (range: 0–36 months). 

The main seizure type at onset is generalized (8/19, 42.1%). Among patients with generalized seizures at onset, four (4/8, 50%) had tonic–clonic seizures, two had absence seizures (typical in one case and atypical in one), one had myoclonic seizures, and one myoclonic–atonic seizures. Six (6/19, 31.6%) patients presented with febrile seizures and three (3/19, 15.8%) had status epilepticus at onset. Two patients (10.5%) had focal seizures (motor in both).

##### Electroencephalogram Pattern (EEG) at Onset

Six (6/19, 31.6%) patients had normal EEG at onset. Excess slow background activity was present in two (2/19, 10.5%) patients. Focal interictal epileptiform discharges were present in one (1/19, 5.3%) patient. Generalized discharges were seen in two patients (2/19, 10.5%), and multifocal discharges were seen in two (2/19, 10.5%). One (1/19, 5.3%) patient had non-convulsive status epilepticus at onset. Data on EEG at onset were not available in five (5/19, 26.3%) patients.

##### Neurological Examination

Eleven (11/19, 57.9%) patients had a normal neurological examination. Eight (8/19, 42.1%) had an abnormal neurological examination (of whom three were ataxic, two had a spastic tetraparesis, and one had strabismus, and in two cases this was not specified). 

##### Neurodevelopmental Features and Psychiatric Comorbidities

Developmental delay/ID was present in eight (8/19, 42.1%) patients. Four (4/19, 21%) patients experienced speech delay, with one case evolving into a specific learning disability. One patient had developmental regression.

Eight (8/19, 42.1%) patients had behavioural and/or psychiatric comorbidities, including aggressive behavior, ASD or autistic traits, and there was inattention in two patients. Obsessive traits, hyperactivity, ideomotor slowing, and irritability were present in one patient each. These impairments were combined in two cases.

##### Neuroimaging Findings

Eleven (11/19, 57.9%) patients had normal brain MRI findings. White matter involvement was present in four (4/19, 21%) cases (periventricular leucomalacia in one, unspecific in three). Two (2/19, 10.5%) patients had a malformation, with a suspected polymicrogyria in one and hypoplastic cerebellum and corpus callosum in the other. Cerebellar atrophy was present in two (2/19, 10.5%), in one case existing with associated cerebral atrophy. 

##### Genetic Testing 

For most patients (13/19, 68%), NGS techniques (i.e., single-gene sequencing, gene panel and exome) were performed. For 3/19 (16%), Sanger sequencing identified the diagnostic variant, while Array-CGH was used in 3 other patients (16%).

**Table 7:** Characteristics of patients carrying a VUS.

Patient	Gender	VUS	Inheritance	Family History	Age at First Seizure	Seizure Type at Onset	DD/ ID	Neurological Examination	Behavioural Problems	EEG Pattern	Brain Nuroimaging	Drug Resistance	Seizures at Last Follow-Up
(1)	F	**46,XX,del(16)(p13.3)** (301 Kb deletion)	Inherited from asymptomatic mother	Negative	36 months	Atypical absences	Present	Normal	No	*At onset*: NA	Normal	No	Seizure-free on oxcarbazepine and levetiracetam
										*At last follow-up*: slow activity			
(2)	F	***CLCN2* [NM_004366.6]:** c.1783T>C: p.Cys595Arg	Inherited from mother, VUS	Negative	15 months	Complex febrile seizure	Present	Ataxic gait and tremor	Yes (aggressive behaviour)	*At onset*: NA	Aspecific abnormalities: hyperintensity of right occipital cortex	Yes	Focal motor seizures
		*COL4A3BP* [NM_001379029.1]: c.979+7T>C	Inherited from mother, likely benign							*At last follow-up*: diffuse abnormalities (mainly in the left temporal region)			
		*SLC9A6* [NM_001379110.1] c.37C>T, p.Arg13Cys	Inherited from father, benign										
(3)	F	***SCN8A*** [NM_001330260.2]: c.4697C>T, p.Thr1566Ile	*de novo*	Negative	36 months	Status epilepticus	No	Normal	No	Focal discharges (frontal)	Normal	No	Seizure-free on carbamazepine
(4)	M	248 Kb **46,XY,del(16)(p13.2)** involving *A2BP1* gene	NA	Negative	13 months	Generalized tonic clonic seizure	Yes (moderate ID)	Macrocephaly, ataxic gait, dysmetria	Present	*At onset*: NA	Hypoplasia of cerebellum and corpus callosus	No	Seizure-free on carbamazepine, valproate and levetiracetam
										*At last follow-up*: slow background activity with sharp waves over posterior regions			
(5)	M	***MTOR*** [NM_004958.3] c.4472G>T, p.Gly1491Val	NA	Positive	24 months	Childhood absence epilepsy (GGE)	Speech delay and specific learning difficulties	Attention deficit and obsessive trait	Normal	*At onset*: Diffuse discharges induced by hyperpnea	Normal	No	Seizure-free on valproate
										*At last follow-up*: no abnormalities			
(6)	F	***SIK1*** [NM_173354.5]: c.718C>T, p.Arg240Cys	Inherited from her mother	Positive	23 months	Generalized tonic–clonic	Normal until 23 months old, then developmental regression	Abnormal	Autistic traits, stereotypies	*At onset*: Several diffuse abnormalities upon falling asleep	Brain MRI: aspecific white matter changes	Yes	Absence seizures
										*At last follow-up*: disorganization of background activity with diffuse discharges			
(7)	F	***WDR45*** [NM_007075.3]:	Mother: negative	Negative	36 months	Myoclonic atonic	Speech delay	Normal	Inattention traits	*At onset*: normal	Brain MRI: normal	Yes	Generalized tonic–clonic seizures
		c.1078G>T, p.(Asp360Tyr)	Father: not performed							*At last follow-up*: slow activity and multifocal discharges			
(8)	M	**46,XY,del(22)(q11.21)**	Inherited from his father	Negative	13 months	Complex febrile seizures and focal motor seizures	Yes	Abnormal	No	*At onset*: multifocal left discharges	Brain MRI: aspecific white matter changes, microcalcifications and suspected polymicrogyria	No	Seizure-free on carbamazepine
										*At last follow-up*: multifocal left discharges			
(9)	M	***GRIN2A:*** [NM_001134407.3]: c.459G>C, p.Gln153His	NA	Negative	Neonatal period	Status epilepticus with recurrent focal motor seizures	Speech delay	Normal	No	*At onset*: frequent theta-delta activity over left fronto-central regions.	Brain MRI: normal	Yes	Focal motor seizures
										*At last follow-up*: multifocal discharges upon falling asleep			
(10)	F	***KCNMA1*** [NM_001161352.1]: c.413C>T, p.Ala138 Val	Both parents are heterozygous for variant	Negative	24 months	Febrile seizures	ID with absent speech	Normal	Ideomotor slowdown, aggressiveness, and irritability	*At onset*: normal	NA	Yes	Atypical absence, tonic and focal motor seizures.
										*At last follow-up*: pattern Lennox–Gastaut			
(11)	M	***HUWE1*:** [NM_031407.7]: c.413C > T, p.Ala138Val	Inherited from his mother	Negative	30 months	Febrile seizures	Speech delay	Normal	Yes (hyperactivity)	*At onset*: normal	Brain MRI: normal	Yes	Focal motor seizures
										*At last follow-up*: theta-delta activity with multifocal discharges			
(12)	F	1. ***SCN1A*:** [NM_001165963.4]: c.419C>T, p.Thr140Ile	NA	Negative	1 month	Myoclonic	Yes	Spastic tetraparesis	No	*At onset*: NA	Brain MRI: progressive cerebral and cerebellar atrophy	Yes	Focal motor seizures
		2. ***HDAC4*** [NM_001378414.1] c.928C>A, p.Val310Ile								*At last follow-up*: slow and disorganized background activities			
(13)	M	***HCN1***: [NM_021072.4]: c.1232A>G, p.Tyr411Cys	Inherited from his father	Positive	14 months	Generalized tonic–clonic seizures with and without fever	No	Normal	No	*At onset*: normal	Normal	No	Seizure-free without therapy
										*At last follow-up*: Multifocal abnormalities with secondary generalization			
(14)	M	1. ***DOCK3*** [NM_004947.5]: c.3884G>A, p.Arg1295Gln	Inherited from his mother	Positive	16 months	Status epilepticus	No	Strabismus	No	*At onset*: non-convulsive status epilepticus	Normal	No	Seizure-free on valproate
		2. ***DOCK3*** [NM_004947.5]: c.5500+6G>A								*At last follow-up*: normal			
(15)	M	***SCN2A*** [NM_001040142.2] c.3385G>	Inherited from his father	Negative	5 months	Focal motor seizures	No	Normal	No	*At onset*: slow activity	Normal	No	Seizure-free
										*At last follow-up*: normal			
(16)	M	***SCN1A*** [NM_0011659634]: *c.99G>C* (*K33N*)	Inherited from his mother	Positive	28 months	Febrile seizures	No	Normal	No	*At onset*: Normal	Normal	No	Seizure-free
										*At last follow-up*: normal			
(17) (HSP)	M	***SCN1A*** [NM_0011659634]: (c.5717T>A) p.I1906N	Not reported	Not reported	5 months	Febrile seizures	No	Normal	No	*At onset*: Normal	Normal	No	Seizure-free
										*At last follow-up*: normal			
(18) (HSP)	F	**46,XXdel(16)(p13.11)**	Not reported	Positive	First days of life	Generalized tonic–clonic seizures	Present	Spastic tetraparesis	No	*At onset*: Not reported	Periventricular leucomalacia	Yes	Focal motor seizures
										*At last follow-up*: disorganization of background activity with focal discharges			
(19)	M	**46,XY,dup(2p21)** and **46,XY,dup(16p13.3)**	Not reported	Positive	6 months	Clonic seizures	Present	Ataxia gait	Yes (autistic spectrum disorder)	*At onset*: Multifocal anomalies	Cerebellar atrophy	yes	Not reported
										*At last follow-up*: slow activity with multifocal discharges upon falling asleep			

##### Seizure Outcome 

Nine (9/19, 47.4%) patients were drug-resistant. Six were seizure-free on therapy, one was seizure-free without therapy, while for three seizure-free patients the information regarding whether they were or not on medications was not available. Drug-resistant patients mainly experienced focal motor seizures (5/19, 31.6%), while generalized seizures (absences in one, tonic–clonic seizures in one) were less represented (2/19, 10.5%). One patient with Lennox–Gastaut syndrome experienced multiple seizure types (atypical absences, tonic and focal motor seizures). The seizure type at the latest follow-up visit was not reported in one patient.

##### EEG Pattern at the Latest Follow-Up Visit

EEG at the end of follow-up revealed disorganized background activity with/without focal or generalized discharges in three patients (3/19, 15.8%), slow background with multifocal interictal discharges in three (3/19, 15.8%), slow background with focal discharges in one (1/19, 5.3%), and a Lennox–Gastaut pattern in one (1/19, 5.3%). Focal interictal discharges were detected in one (1/19, 5.3%), with multifocal discharges in three patients (3/19, 15.8%). Five (5/19, 26.3%) patients had a normal EEG.

## 3. Discussion

We are reporting on a retrospective multicenter Italian cohort of patients that had their onset of genetic epilepsies or developmental and epileptic encephalopathies within the first three years of life. We aimed to better define their electroclinical, neuroimaging, and genetic profiles, their epilepsy outcomes at the end of the follow-up, and the occurrence of neurodevelopmental and psychiatric comorbidities.

Although our study design included patients with epilepsy onset within 36 months of age, the mean age of onset is significantly lower (11 months), reflecting previous literature data [24,25,26] and the decline of diagnostic yield of genetic testing with increasing age at epilepsy onset [27,28,29].

In our cohort, the most represented seizure type at onset is generalized, especially in infants and children with CNVs and chromosomopathies (62% versus 37.5% in children with monogenic conditions). Previous studies [30,31] also reported on the prevalence of generalized seizures, the most common within the group being either tonic–clonic seizures [30] or epileptic spasms [31]. Interestingly, we found that the distribution of seizure types at onset is different between patients with monogenic conditions and patients harbouring a CNV or chromosomal abnormality, with focal seizures being more than three times more common in monogenic conditions, while the frequency of febrile seizures, focal-to-bilateral seizures, and status epilepticus at onset is similar in the two groups.

We also documented very high figures of abnormal neurological examination in both subgroups. We believe that this strongly confirms that, differently from older age groups, early-onset genetic epilepsies often occur in the context of complex neurological phenotypes, in which epilepsy is just one of many dynamic clinical targets needing to be addressed with a holistic approach. In particular, the association of epilepsies and DEE with movement disorders is gaining increasing attention in the literature. In a recent paper analyzing a single centre’s experience in the follow-up of persons with monogenic conditions and clinically affected by epilepsy and movement disorder, the investigators found that, in their sample, the semiology of movement disorders (especially the presence of hypokinetic versus hyperkinetic movement disorders) tended to identify two aetiologically different groups: the first mainly involving neurodegenerative conditions and the second mainly involving defects of neurotransmission, neuronal excitability, or neural development [32]. However, this finding should not be interpreted in absolute terms, as it must be noted that hyperkinetic movement disorders (such as ataxia or spasticity) are well-described features of various neurodegenerative disorders [33,34]. Additional relevant phenotypic clusters in our cohort include hereditary spastic paraplegias (HSP). Within complex HSP cases, epilepsy is found in a relevant subset of pediatric-onset cases [35]. Thus neurological features, together with the epilepsy phenotype, can represent useful handles to formulate the correct diagnostic hypotheses.

In line with these observations, we documented heterogeneous neuroimaging features, which can be divided into three main groups: normal, aspecific and abnormal. According to a study performed on an unselected cohort of children with new-onset epilepsy starting before 3 years of age, aetiologically relevant findings were present in 40% and incidental findings in an additional 15% of patients [24]. In our series, normal neuroimaging findings prevailed in children with monogenic conditions, while the majority of patients with CNVs or chromosomal aberrations had abnormal neuroimaging. Patients with monogenic conditions had malformations in 32 cases and progressive MRI changes in 16, while in the group with CNVs and chromosomal aberrations the ratio was 21/4. We found typical brain MRI findings (i.e., cortical malformations in *TUBB*-related disorder or lissencephaly with a pathogenic *LIS1* variant), but also aspecific findings. Importantly, the presence of progressive MRI changes, such as cerebral or cerebellar atrophy, identified a subgroup of children for whom receiving neuroimaging is even more critical for correct management and diagnosis. In some cases, neuroimaging features pointed out overt neurodegenerative conditions (i.e., large subcortical cysts in megalencephalic leukoencephalopathy with subcortical cysts), thus informing further investigations into neurogenetic and neurometabolic disorders.

Unsurprisingly [36,37,38], comorbidities with neurodevelopmental and psychiatric disorders were common. The vast majority of patients had DD or ID (80.7% of individuals with monogenic conditions and 91% of those with CNVs or chromosomal abnormalities), and autism spectrum disorder was diagnosed in 11.5% of individuals with monogenic conditions and in one person with a CNV. Behavioural issues/psychiatric disorders were more common in those harbouring CNVs or chromosomal abnormalities than in monogenic conditions (33.3% versus 14.4%). The associations we documented have been well established in the literature: 1p36 deletion syndrome and abusive/aggressive behaviour [39], *PCDH19* pathogenic variants and hyperactive, autistic, and obsessive-compulsive features [40], *CHD2* pathogenic variants and hyperactivity [41], *MBD5* and limited social interactions, aggressive and self-injurious behavior, short attention span, and autistic features [42]. Although the prevalence of behavioural and psychiatric comorbidities generally increases in persons with intellectual disability [43], growing evidence supports the view that the link between epilepsy and neurobehavioral impairments is based on specific neurobiological mechanisms [44], including changes in neurotransmitters/neuromodulators, hypothalamic–pituitary adrenal axis dysfunction, network dysfunction, altered neurogenesis, neurotrophic factors, and neuroinflammation [45]. The complex relationship between epilepsy and its neurobehavioural comorbidities is further suggested by one retrospective observational study in Norway, highlighting how the prevalence of these comorbidities is similar in focal and generalized epilepsies, but significantly higher in focal epilepsy of unknown cause compared to lesional epilepsy, and independent of seizure control [46]. This might suggest a more critical role for intrinsic (genetically based) susceptibility factors. This hypothesis is also corroborated by the lower age of seizure onset in persons with focal epilepsy with comorbidities compared to those without [46]. 

At the end of the follow-up period, drug resistance occurred slightly more frequently in individuals with monogenic epilepsies than in those harbouring CNVs or chromosomal abnormalities, and 42% versus 58% of patients were seizure-free at their latest evaluation. In a population-based study on patients with early-childhood-onset epilepsy, 28% were drug-resistant, of whom 47% had monogenic epilepsy [30].

DEE- and epilepsy-related genes can be grouped into five categories: ion transport; cell growth and differentiation; regulation of synaptic processes; transport and metabolism of small molecules; and regulation of gene transcription and translation [47,48]. Among monogenic disorders, the largest group in our cohort includes children harbouring P/LP variants in genes encoding ion channels (30%), in line with previous research [47,49,50]. However, 28 genes were involved on only one occasion, strikingly highlighting the vast genetic heterogeneity underlying early-onset epilepsies and DEE [51]. 

Even if it is well known that the association between genomic disorders and epilepsy varies in terms of prevalence and semiology, and that in syndromic epilepsies seizures are part of a multisystem abnormality, with different types of potentially associated seizures [38], we decided to include patients presenting with CNVs and chromosomal abnormalities. We based our choice on the presence of epilepsy-related genes inside the deleted/duplicated regions, of documented enrichment in epilepsy, or on their relationship to genetic OMIM syndromes featuring neurological symptoms, including epilepsy [52]. Our results are in line with the literature in that the most common CNVs include 1p36 deletion syndrome [53] and rearrangements involving chromosome 16 [54].

In our cohort, we documented a higher percentage of DEE in those with monogenic conditions (54% versus 38%). This is in agreement with a previous observation that, when epilepsy manifests as DEE, it is more likely to be caused by pathogenic variants in single genes rather than by CNVs [52].

After careful diagnostic work-up and re-evaluation of clinical and genetic reports and variants classification, the detected genetic variations had an uncertain clinical significance in 11% of cases. This was lower than in a recently published pediatric cohort in which 16.4% of tested patients had at least one VUS detected with chromosomal microarray and 41.9% via NGS sequencing of a panel of epilepsy-related genes [55]. However, study design was different from ours.

For each and every involved gene, we confirmed that the phenotypic spectrum was very wide. We documented some clinical features partially, rarely, or never described in the literature (Supplementary Table S1). Two sisters carrying a compound heterozygous variation in the *ALDH18A1* gene presented with the so-far-unreported phenotype of DEE-SWAS in the context of spastic paraplegia. In fact, to the best of our knowledge, only one patient with complex spastic paraplegia featuring epilepsy has been described, but he experienced temporal lobe seizures [56]. Among patients harbouring *SCN1A* LP/P variants, although the largest group was composed of patients with Dravet syndrome followed by those with a GEFS plus phenotype [57,58,59], we also reported on one patient [12] with the recently defined phenotype of neonatal developmental and epileptic encephalopathy with movement disorders and arthrogryposis, associated with gain-of-function *SCN1A* variants [60]. One female patient carried a pathogenic *ARHGEF9* single-gene variant, which was an atypical finding because females are usually healthy carriers and few descriptions of affected subjects are available [18]. Furthermore, in one patient with atypical Rett syndrome, we found a pathogenic mosaic variant in the *GABRG2* gene, which is usually associated with different epilepsy phenotypes but has not been reported elsewhere in association with Rett syndrome [17]. A female patient harbouring an LP variant in the *PIGW* gene with early-onset epilepsy and a complex neurological phenotype achieved seizure control in late childhood. She is currently the oldest known patient out of a total of 7 published worldwide [15,61]. A final patient with Snyder–Robinson syndrome (secondary to a pathogenic hemizygous *SMS* gene variant) had myoclonic seizures, which have been reported in only one additional patient [62]. We think that such cases are good examples of the role of NGS technologies (and especially ES) in solving atypical, unusual, or complex phenotypes. Reaching a precise and timely genetic diagnosis is important in order to correctly define the recurrence risk, and (when applicable) to aim for a targeted therapy [63,64,65,66].

Our study had several limitations. Due to the retrospective design, in some cases we were unable to retrieve all the relevant information for each patient. Furthermore, diagnostic tests were selected at the discretion of the treating physician and not as part of a trial, although evidence-based international recommendations were followed. Finally, we did not perform a statistical analysis of our data, but rather qualitatively described our findings. 

However, we think that there are also some strengths to this work, such as the collection of detailed clinical, EEG, neuroimaging, and genetic data over a mean follow-up period of 14.75 years. Data analysis involved both clinical geneticists and pediatric neurologists at each of the four collaborating centres.

## 4. Materials and Methods

This retrospective observational cohort study was carried out at four Italian epilepsy centers (Epilepsy Center of San Paolo University Hospital in Milan, Child Neurology and Psychiatry Unit of AUSL-IRCCS di Reggio Emilia, Pediatric Neurology Unit of Vittore Buzzi Children’s Hospital, Milan, and Child Neurology and Psychiatry Unit, IRCCS Mondino Foundation, Pavia). 

### 4.1. Inclusion Criteria

Inclusion criteria were as follows: (a) genetic epilepsies with pathogenic or likely pathogenic variants and VUS; (b) age of epilepsy onset in the first three years of life.

The choice to include children with epilepsy onset within 36 months of age was made because the risk of cognitive impairment, behavioral comorbidities, and drug resistance was higher in this age group [67].

### 4.2. Exclusion Criteria

Exclusion criteria were as follow: (a) epilepsies related to other aetiological causes (such as inborn metabolic diseases and acquired structural aetiologies); (b) patients with a tuberous sclerosis complex and a typical Rett syndrome harbouring pathogenic variants on the methyl-CpG binding protein 2 (*MECP2*) gene. This choice was made to ensure better homogeneity of the sample because the Epilepsy Centre of San Paolo University Hospital in Milan has been a reference centre for these two diseases since 2006–2007. 

### 4.3. Data Collection 

Detailed clinical features were retrospectively collected by reviewing medical charts, consultations reports, and discharge letters. Apart from reading and annotating the reports, neuroimaging and electroencephalogram (EEG) data were directly reviewed. All data were gathered in a database.

Informed consent for genetic testing was obtained from all children’s parents. For this paper, a formal approval from the local ethics committee was waived because we retrospectively reported on observational data.

For each patient, information about the following variables were collected: gender, family history for epilepsy and/or febrile seizures, epileptic features, neurologic examination, cognitive impairments and behavioral issues, neuroimaging features, metabolic and genetic findings. 

Regarding the epileptic phenotype, we evaluated age at epilepsy onset, type of seizures at onset and at the last follow-up, EEG pattern at onset and at the latest follow-up, drug therapy, and drug resistance. 

We classified seizure types according to the 2017 ILAE Classification [1] and epilepsy syndromes according to the 2022 ILAE Classification and definition of epilepsy syndromes with onset in childhood [68]. Moreover, we categorized genetic variants according to the guidelines and recommendations of the American College of Medical Genetics and Genomics (ACMG) [69].

Brain magnetic resonance imaging or computed tomography were performed according to clinical presentation at the discretion of the treating physician. The presence of any acquired structural abnormalities was an exclusion criterion. 

Furthermore, we evaluated genetic consultations and assessed which genetic test led to diagnosis for each patient. Performed genetic tests include karyotype, CGH-array, single-gene Sanger sequencing or multiplex ligation-dependent probe amplification (MLPA), and NGS (targeted gene panels, ES or GS). NGS results were all confirmed by Sanger sequencing [70]. The specific genetic test result was considered as diagnostic based on a thorough evaluation by the multidisciplinary team of pediatric neurologists and clinical geneticists at each participating center, and according to well-established guidelines and recommendations [71]. The selection of the genetic test (s) to administer to each patient was made by the treating physician, on a clinical basis, according to current evidence and best clinical practice [71]. The functional role of detected genes was categorized based on [48,72,73].

Psychomotor and cognitive development was evaluated by formal neuropsychological testing (such as Griffiths Mental Development Revised Scales [74], Wechsler Preschool and Primary Scale of Intelligence—WPPSI [75], Wechsler Intelligence Scale for Children—WISC) [76,77] or, if unavailable, best clinical assessment (based on developmental milestones and academic achievements) [78,79]. 

We divided our patients’ cohort into two groups: patients with pathogenic and likely pathogenic variants and patients with VUS. Within the group of patients with pathogenic and likely pathogenic genetic variations, we further distinguished between monogenic conditions and chromosomal abnormalities (copy number variations—CNV—and structural defects). In the subgroup with monogenic conditions, we also included microdeletions containing genes known to be associated with diseases, which act with a loss of function mechanism (i.e., 16p11.2 microdeletion syndrome and proline-rich transmembrane protein 2—*PRRT2*—gene) [80,81].

## 5. Conclusions

In conclusion, the main findings in our retrospective multicentre study of genetically caused epilepsies and DEE with onset within the first three years of life are: a high occurrence of generalized seizures at onset, drug resistance, abnormal neurological examination, global developmental delay and intellectual disability, and comorbidities with behavioural and psychiatric issues. We also documented different presentations between monogenic versus CNVs and chromosomal conditions, and atypical or rare phenotypes. A subgroup of patients with progressive neuroimaging changes highlighted how the diagnostic work-up and clinical management of early-childhood epilepsies can significantly diverge from that of older age groups and be more complex.

## Figures and Tables

**Table 1:** Cell functions of causative genes.

Function	Gene Name
Ion channels	Sodium voltage-gated channel alpha subunit 1 (*SCN1A*) Sodium voltage-gated channel alpha subunit 8 (*SCN8A*) Sodium channel, voltage-gated, type II, alpha subunit (*SCN2A*) Potassium voltage-gated channel subfamily Q member 2 (*KCNQ2*) Potassium sodium-activated channel subfamily T member 1 (*KCNT1*) Calcium voltage-gated channel subunit alpha1 G (*CACNA1G*)
Enzymes	Cyclin-dependent kinase-like 5 (*CDKL5*) Chromodomain helicase DNA-binding protein 2 (*CHD2*) Lissencephaly 1 (*LIS1*) or platelet-activating factor acetylhydrolase 1b regulatory subunit 1 (*PAFAH1B1*) Aldehyde dehydrogenase 18 family member A1 (*ALDH18A1*) Adenylosuccinate lyase (*ADSL*) Mechanistic target of rapamycin (*MTOR*) Arginyl-tRNA synthetase 2, mitochondrial (*RARS2*) Inhibitor of nuclear factor kappa B kinase regulatory subunit gamma (*IKBKG*) Ribosomal protein S6 kinase A3 (*RPS6KA3*) Spermine synthase (*SMS*) Glycine decarboxylase (*GLDC*) Polynucleotide kinase 3′-phosphatase (*PNKP*) Molybdenum cofactor synthesis 1 (*MOCS1*) Molybdenum cofactor synthesis 2 (*MOCS2*) Phosphatidylinositol glycan anchor biosynthesis class W (*PIGW*) Cdc42 guanine nucleotide exchange factor 9 (*ARHGEF9*)
Receptors	Gamma-aminobutyric acid type A receptor subunit alpha 1 (*GABRA1*) Gamma-aminobutyric acid type A receptor subunit gamma2 (*GABRG2*)
Cell adhesion molecules	Protocadherin-19 (*PCDH19*)
Synaptic function	Proline-rich transmembrane protein 2 (*PRRT2*) IQ motif and Sec7 domain 2 (*IQSEC2*) Synaptic Ras GTPase-activating protein 1 (*SYNGAP1*) Syntaxin-1B (*STX1B*)
Trafficking	Syntaxin-binding protein 1 (*STXBP1*) Phosphofurin acidic cluster sorting protein 1 (*PACS1*) RAB39B member RAS oncogene family (*RAB39B*)
Transcription factors	Forkhead box G1 (*FOXG1*) GATA binding protein 3 (*GATA3*)
Transcriptional regulators	Methyl-CpG binding domain protein 5 (*MBD5*)
DNA binding	Lysine (K)-specific methyltransferase 2A (*KMT2A*) Heterogeneous nuclear ribonucleoprotein U (*HNRNPU*)
Transporters	Solute carrier family 2 (facilitated glucose transporter), member 1 (*SLC2A1*) Solute carrier family 19 member 3 (*SLC19A3*)
ATPase	ATPase Na+/K+-transporting subunit alpha 3 (*ATP1A3*)
Autophagy	WD repeat domain 45 (*WDR45*)
Cell proliferation/apoptosis	SAM and HD domain containing deoxynucleoside triphosphate triphosphohydrolase 1 (*SAMHD1*)
Cell junction	Megalencephalic leukoencephalopathy with subcortical cysts 1 (*MLC1*)
Structural component of microtubules	Tubulin beta class I (*TUBB*)
Microtubule-associated proteins	WD repeat domain 62 (*WDR62*)
Cytoskeleton	Glial fibrillary acidic protein (*GFAP*)
Intracellular signaling	Seizure threshold 2 (*SZT2*)

**Table 2:** Neurological examination findings in patients with monogenic disorders.

Neurological Examination	Genes
Normal	*PRRT2*, *KCNQ2*, *SCN1A*, *SCN1B*, *SCN8A*, *PCDH19*, *RAB39B*, *STX1B*, *SCN2A*, *CHD2*, *KCNB1*, *KCNQ3*, *IQSEC2*, *PACS1*
Macrocephaly	*HNRNPU*
Microcephaly	*CDKL5*, *ATP1A3*, *PNKP*, *KCNT1*
Hypotonia	*RPS6KA3*, *CDKL5*, *MTOR*, *PIGW*, *SYNGAP1*, *KCNQ2*, *SZT2*, *ATP1A3*, *SCN1A*, *SAMHD1*, *GLDC*, *MBD5*
Spastic tetraparesis	*CDKL5*, *LIS1*, *WDR62*, *HNRNPU*, *MOCS1*, *RARS2*, *KMT2A*, *GFAP*, *STXBP1*, *SCN8A*, *PNKP*, *KCNT1*, *ADSL*, *KCNQ2*
Hemiparesis	*MOCS2*
Spastic paraplegia	*ALDH18A1*
Pyramidal signs	*SLC2A1*, *STXBP1*, *KCNQ2*, *SCN2A*, *CACNA1G*,*MLC1*
Gait abnormalities (including ataxia)	*RPS6KA3*, *ARGHEF9*, *GABRG2*, *KCNT1*, *SCN1A*, *MTOR*, *STXBP1*, *PIGW*, *CACNA1A*, *TUBB*, *SMS*, *GABRA1*, *MLC1*
Extrapyramidal signs	*SCN1A*
Movement disorders	*WDR45* (hand stereotypies), *FOXG1* (dystonia), *GABRG2* (hand stereotypies), *KCNT1* (hand stereotypies), *SCN1A* (*tics*), *PIGW* (hand stereotypies), *KCNQ2* (hand stereotypies), *SZT2* (hand stereotypies), *SLC2A1* (dyskinesia), *STXBP1* (dystonia), *GLDC* (hand stereotypies)
Disorders of the visual system	*PCDH19* (ptosis), *TUBB*
Disorders of ocular motility	*WDR45* (strabismus), *GATA3* (strabismus), *HNRNPU*, *KCNQ2* (strabismus and nystagmus)
Skin hyperpigmentation	*IKBKG*
Hearing loss	*GATA3*
Congenital clubfoot	*SCN1A*

**Table 3:** Psychiatric and behavioural comorbidities in patients with monogenic disorders. “+” means: “present”.

	ASD	ADHD	Irritability and Psychomotor Agitation	Attachment Disorder	OCD	Psychotic Disorders
*WDR45*			+			
*MBD5*		+	+			
*KCNQ2*	+					
*CACNA1G*	+					
*GLDC*			+			
*ADSL*	+					
*PACS1*				+		
*KCNQ2*	+					
*PNKP*	+					
*STXBP1*	+					
*MOCS1*			+			
*PCDH19*	+	+			+	+
*CHD2*		+				
*SYNGAP1*			+			
*HNRNPU*	+					
*GATA3*			+			
*STX1B*			+			
*RAB39B*			+			
*SCN1A*	+		+			+
*MTOR*	+					
*PRRT2*		+				
*GABRG2*			+			
*FOXG1*			+			

**Table 4:** Brain MRI findings in monogenic disorders.

Brain MRI Findings	Genes
Normal	*GABRA1*, *CACNA1G*, *KCNT1*, *SCN2A*, *STXBP1*, *SCN1B*, *SLC2A1*, *PCDH19*, *PRRT2*, *KCNQ3*, *CHD2*, *SYNGAP1*, *KCNB1*, *ATP1A3*, *HNRNPU*
Cerebral atrophy	*WDR45*, *SLC19A3*, *ADSL*, *MOCS1*, *SCN1A*, *GABRA1*, *PIGW*, *KCNT1*, *CDKL5*, *FOXG1*
Cerebellar atrophy	*WDR45*, *SLC19A3*, *ADSL*, *CACNA1A*, *SCN1A*
Cerebellar hypoplasia	*RARS2* (pons)
Corpus callosum dysgenesis	*WDR45*, *SMS*, *KCNQ2*, *KMT2A*
Corpus callosum hypoplasia	*SZT2*
Malacic lesions	*IKBKG*
Periventricular white matter changes	*RPS6KA3* (Coffin–Lowry syndrome)
Hypointense signal in the substantia nigra and globus pallidus	*WDR45*
Large subcortical cysts	*MLC1*
Ventricular dilatation (ventriculomegaly)	*SMS*
Cerebrospinal fluid space enlargements	*ALDH18A1*, *CDKL5*, *STXBP1*, *PRRT2*, *CDKL5*
Optic nerves thinning	*TUBB*
Malformations of cortical development	*TUBB*, *MTOR*, *WDR62* (schizencephaly), *LIS1*, *PNKP*
White matter abnormalities (including hypomyelination)	*SLC19A3*, *KCNT1*, *SCN8A*, *GFAP*, *SAMHD1*, *KMT2A*, *PCDH19*, *SZT2*, *KCNQ2*, *CDKL5*
Large cisterna magna	*KCNQ2*
Diffusion restriction in the posterior limb of the internal capsule	*GLDC*
Basal Ganglia involvement	*MOCS*, *GFAP*, *FOXG1* (lacunar infarct)
Brain Calcification	*SAMHD1*
Mesial temporal sclerosis	*ATP1A3*
Hydromyelia	*ARGHEF9*

**Table 5:** Neurological examination findings in patients with chromosomal abnormalities and CNVs.

Neurological Examination	Chromosomal Abnormalities
Normal	46,XX,dup(14)(q11.2q12), 46,XX,dup(16)(p13.11p12.3), 46,XY,del(16)(p11.2)
Microcephaly	Angelman syndrome, 1p36 terminal deletion syndrome, 46,XX,del(9qter), 46,XX,del(8)(p23.3p23.2) and 46,Xxdup(13)(q32.1q34), 46,XY,del(6)(q26-qter)
Hypotonia	Trisomy 21, Angelman syndrome, InvDup (15) syndrome, 46,XX,del(9qter), 1p36 terminal deletion syndrome, 46,XX,del(1q44), Xq28 duplication syndrome, 46,XY,del(6)(q26-qter)
Spastic tetraparesis	46,XX,del(4p16.3), Angelman syndrome, Xq28 duplication syndrome, 1p36 terminal deletion syndrome, 46,XY,del(17p13.3), 46,XX,del(9)(q33.3q34.11), trisomy 13
Pyramidal signs	InvDup(15) syndrome, 46,XX,del(1q44)
Movement disorders	Angelman syndrome (tremulousness of the limbs), InvDup (15) syndrome (hand stereotypies), 46,XX,del(5)(q11.2q13.2) and 46,XX,dup(5q13.2) (hand stereotypies), 1p36 terminal deletion syndrome (hand stereotypies)
Gait abnormalities (including ataxia)	Angelman syndrome, 1p36 terminal deletion syndrome, 46,XX,del(9qter), 46,XX,del(6)(q21q22.31), 46,XY,del(6)(q26-qter)
Dyspraxia	del 17q21.31
Disorders of ocular motility	46,XX,del(5)(q11.2q13.2) and 46,XX,dup(5q13.2) (strabismus and nystagmus), 1p36 terminal deletion syndrome, 46,XX,del(1q44), 46,XY,del(6)(q26-qter) (nystagmus)
Hearing loss	46,XX,del(8)(p23.3p23.2) and 46,Xxdup(13)(q32.1q34)
Scoliosis	Angelman syndrome

**Table 6:** Brain MRI findings in chromosomal abnormalities and CNVs.

Brain MRI Findings	Chromosomal Abnormalities
Normal	46,XY,del(2)(q24.2q24.3), 46,XY,del(16p11.2), 46,XX,dup(15q11.2) 46,XX,del(16p11.2), 46,XX,dup(14)(q11.2q12, 46,XX,del(4p16.3), 46,XX,del(8)(p23.3p23.2) and 46,Xxdup(13)(q32.1q34), 46,XX,del(6)(q21q22.31), Angelman syndrome
Cerebellar atrophy	Wolf–Hirschhorn syndrome
Cerebellar hypoplasia	Trisomy 21 (vermis), Wolf–Hirschhorn syndrome, Xq28 duplication syndrome
Corpus callosum dysgenesis	Angelman syndrome (dysmorphic), trisomy 13, 46,XX,del(1q44)
Cerebrospinal fluid space enlargements	Trisomy 21
Enlarged subarachnoid spaces	Xq28 duplication syndrome, InvDup(15) syndrome
Malformations of cortical development	46,XY,del(6)(q26-qter), 46,XY,del(17p13.3)
White matter abnormalities (including hypomyelination)	Trisomy 21, 1p36 terminal deletion syndrome, Angelman syndrome, 46,XX,del(9qter)
Basal ganglia involvement	46,XX,del(9qter)
Vascular lesion due to Takayasu arteritis	Wolf–Hirschhorn syndrome

## Data Availability

The data supporting the findings of this study are available on request from the corresponding author. The data are not publicly available due to privacy restrictions.

## Supplementary Materials

The following supporting information can be downloaded at: https://www.mdpi.com/article/10.3390/ijms25021248/s1.

## Abbreviations

ACMG: American College of Medical Genetics and Genomics; ADHD: attention deficit hyperactivity disorder; *ADSL*: adenylosuccinate lyase; *ALDH18A1*: aldehyde dehydrogenase 18 family member A1; *ARHGEF9*: Cdc42 guanine nucleotide exchange factor 9; *ATP1A3*: ATPase Na+/K+-transporting subunit alpha 3; *CACNA1G*: calcium voltage-gated channel subunit alpha1 G; *CDKL5*: cyclin-dependent kinase-like 5; CGH-array: comparative genomic hybridization array; *CHD2*: chromodomain helicase DNA-binding protein 2; CNV: copy number variations; CT: computed tomography; DD: developmental delay; DEE: developmental and epileptic encephalopathies; DEE-SWAS: developmental/epileptic encephalopathy with spike wave activation in sleep; EEG: electroencephalogram; ES: epileptic spasms; *FOXG1*: forkhead box G1; *GABRA1*: gamma-aminobutyric acid type A receptor subunit alpha 1; *GABRG2*: gamma-aminobutyric acid type A receptor subunit gamma2; *GATA3*: GATA binding protein 3; *GFAP*: glial fibrillary acidic protein; *GLDC*: glycine decarboxylase; *HNRNPU*: heterogeneous nuclear ribonucleoprotein U; HSP: hereditary spastic paraplegia; ID: intellectual disability; *IKBKG*: inhibitor of nuclear factor kappa B kinase regulatory subunit gamma; ILAE: International League Against Epilepsy; *IQSEC2*: IQ motif and Sec7 domain 2; *KCNQ2*: potassium voltage-gated channel subfamily Q member 2; *KCNT1*: potassium sodium-activated channel subfamily T member 1; *KMT2A*: lysine (K)-specific methyltransferase 2A; *LIS1*: lissencephaly 1; LP: likely pathogenic; *MBD5*: methyl-CpG binding domain protein 5; *MECP2*: methyl-CpG binding protein 2; *MLC1*: megalencephalic leukoencephalopathy with subcortical cysts 1; MLPA: multiplex ligation-dependent probe amplification; MRI: magnetic resonance imaging; *MOCS1*: molybdenum cofactor synthesis 1; *MOCS2*: molybdenum cofactor synthesis 2; MTOR: mechanistic target of rapamycin; NA: not available; NGS: next-generation sequencing; P: pathogenic; *PACS1*: phosphofurin acidic cluster sorting protein 1; *PAFAH1B1*: platelet-activating factor acetylhydrolase 1b regulatory subunit 1; *PCDH19*: Protocadherin-19; *PIGW*: phosphatidylinositol glycan anchor biosynthesis class W; *PNKP*: polynucleotide kinase 3′-phosphatase; *PRRT2*: proline-rich transmembrane protein 2; *RAB39B*: RAB39B member RAS oncogene family; *RARS2*: arginyl-tRNA synthetase 2, mitochondrial; *RPS6KA3*: ribosomal protein S6 kinase A3; *SAMHD1*: SAM And HD domain containing deoxynucleoside triphosphate triphosphohydrolase 1; *SCN1A*: sodium voltage-gated channel alpha subunit 1; *SCN2A*: sodium channel, voltage-gated, type II, alpha subunit; *SCN8A*: sodium voltage-gated channel alpha subunit 8; *SLC2A1*: solute carrier family 2 (facilitated glucose transporter), member 1; *SLC19A3*: solute carrier family 19, member 3; *SMS*: spermine synthase; *STX1B*: syntaxin-1B; *STXBP1*: syntaxin-binding protein 1; *SYNGAP1*: synaptic Ras GTPase-activating protein 1; *SZT2*: seizure threshold 2; *TUBB*: Tubulin Beta Class I; VUS: variants of uncertain significance; *WDR45*: WD repeat domain 45; *WDR62*: WD repeat domain 62; WES: whole-exome sequencing; WGS: whole-genome sequencing; WISC: Wechsler Intelligence Scale for Children; WPPSI: Wechsler Preschool and Primary Scale of Intelligence.

## References

[1] Scheffer I.E., Berkovic S., Capovilla G., Connolly M.B., French J., Guilhoto L., Hirsch E., Jain S., Mathern G.W., Moshé S.L. (2017). ILAE classification of the epilepsies: Position paper of the ILAE Commission for Classification and Terminology. Epilepsia.

[2] Hebbar M., Mefford H.C. (2020). Recent Advances in Epilepsy Genomics and Genetic Testing. F1000Research.

[3] Balestrini S., Arzimanoglou A., Blümcke I., Scheffer I.E., Wiebe S., Zelano J., Walker M.C. (2021). The aetiologies of epilepsy. Epileptic Disord..

[4] Syvertsen M., Nakken K.O., Edland A., Hansen G., Hellum M.K., Koht J. (2015). Prevalence and etiology of epilepsy in a Norwegian county-A population based study. Epilepsia.

[5] Falco-Walter J. (2020). Epilepsy-Definition, Classification, Pathophysiology, and Epidemiology. Semin. Neurol..

[6] Weber Y.G., Biskup S., Helbig K.L., Von Spiczak S., Lerche H. (2017). The role of genetic testing in epilepsy diagnosis and management. Expert Rev. Mol. Diagn..

[7] Rastin C., Schenkel L.C., Sadikovic B. (2023). Complexity in Genetic Epilepsies: A Comprehensive Review. Int. J. Mol. Sci..

[8] Ji J., Leung M.L., Baker S., Deignan J.L., Santani A. (2021). Clinical exome reanalysis: Current practice and beyond. Mol. Diagn. Ther..

[9] Brock D.C., Abbott M., Reed L., Kammeyer R., Gibbons M., Angione K., Bernard T.J., Gaskell A., Demarest S. (2023). Epilepsy panels in clinical practice: Yield, variants of uncertain significance, and treatment implications. Epilepsy Res..

[10] Johannesen K.M., Tümer Z., Weckhuysen S., Barakat T.S., Bayat A. (2023). Solving the unsolved genetic epilepsies: Current and future perspectives. Epilepsia.

[11] Koh H.Y., Smith L., Wiltrout K.N., Podury A., Chourasia N., D’Gama A.M., Park M., Knight D., Sexton E.L., Koh J.J. (2023). Utility of Exome Sequencing for Diagnosis in Unexplained Pediatric-Onset Epilepsy. JAMA Netw. Open.

[12] Spagnoli C., Frattini D., Rizzi S., Salerno G.G., Fusco C. (2019). Early infantile *SCN1A* epileptic encephalopathy: Expanding the genotype-phenotype correlations. Seizure.

[13] Mastrangelo M., Peron A., Spaccini L., Novara F., Scelsa B., Introvini P., Raviglione F., Faiola S., Zuffardi O. (2013). Neonatal suppression-burst without epileptic seizures: Expanding the electroclinical phenotype of STXBP1-related, early-onset encephalopathy. Epileptic Disord..

[14] Peron A., Spaccini L., Norris J., Bova S.M., Selicorni A., Weber G., Wood T., Schwartz C.E., Mastrangelo M. (2013). Snyder-Robinson syndrome: A novel nonsense mutation in spermine synthase and expansion of the phenotype. Am. J. Med. Genet. A.

[15] Peron A., Iascone M., Salvatici E., Cavirani B., Marchetti D., Corno S., Vignoli A. (2020). PIGW-related glycosylphosphatidylinositol deficiency: Description of a new patient and review of the literature. Am. J. Med. Genet. A.

[16] Aspromonte M.C., Bellini M., Gasparini A., Carraro M., Bettella E., Polli R., Cesca F., Bigoni S., Boni S., Carlet O. (2020). Characterization of intellectual disability and autism comorbidity through gene panel sequencing. Hum. Mutat..

[17] Cogliati F., Giorgini V., Masciadri M., Bonati M.T., Marchi M., Cracco I., Gentilini D., Peron A., Savini M.N., Spaccini L. (2019). Pathogenic Variants in *STXBP1* and in Genes for GABAa Receptor Subunities Cause Atypical Rett/Rett-like Phenotypes. Int. J. Mol. Sci..

[18] Scala M., Zonneveld-Huijssoon E., Brienza M., Mecarelli O., van der Hout A.H., Zambrelli E., Turner K., Zara F., Peron A., Vignoli A. (2021). De novo ARHGEF9 missense variants associated with neurodevelopmental disorder in females: Expanding the genotypic and phenotypic spectrum of ARHGEF9 disease in females. Neurogenetics.

[19] Carter L.B., Battaglia A., Cherry A., Manning M.A., Ruzhnikov M.R., Bird L.M., Dowsett L., Graham J.M., Alkuraya F.S., Hashem M. (2019). Perinatal distress in 1p36 deletion syndrome can mimic hypoxic ischemic encephalopathy. Am. J. Med. Genet. A.

[20] Spagnoli C., Salerno G.G., Iodice A., Frattini D., Pisani F., Fusco C. (2018). KCNQ2 encephalopathy: A case due to a de novo deletion. Brain Dev..

[21] Fusco C., Frattini D., Bassi M.T. (2015). A novel KCNQ3 gene mutation in a child with infantile convulsions and partial epilepsy with centrotemporal spikes. Eur. J. Paediatr. Neurol..

[22] Maini I., Iodice A., Spagnoli C., Salerno G.G., Bertani G., Frattini D., Fusco C. (2016). Expanding phenotype of PRRT2 gene mutations: A new case with epilepsy and benign myoclonus of early infancy. Eur. J. Paediatr. Neurol..

[23] Iodice A., Spagnoli C., Frattini D., Salerno G.G., Rizzi S., Fusco C. (2019). Biallelic SZT2 mutation with early onset of focal status epilepticus: Useful diagnostic clues other than epilepsy, intellectual disability and macrocephaly. Seizure.

[24] Coryell J., Gaillard W.D., Shellhaas R.A., Grinspan Z.M., Wirrell E.C., Knupp K.G., Wusthoff C.J., Keator C., Sullivan J.E., Loddenkemper T. (2018). Neuroimaging of Early Life Epilepsy. Pediatrics.

[25] Hunter M.B., Yoong M., Sumpter R.E., Verity K., Shetty J., McLellan A., Chin R.F.M. (2020). Incidence of early-onset epilepsy: A prospective population-based study. Seizure.

[26] Hauser W.A., Annegers J.F., Kurland L.T. (1993). Incidence of epilepsy and unprovoked seizures in Rochester, Minnesota: 1935–1984. Epilepsia.

[27] De Wachter M., Schoonjans A.S., Weckhuysen S., Van Schil K., Löfgren A., Meuwissen M., Jansen A., Ceulemans B. (2023). From diagnosis to treatment in genetic epilepsies: Implementation of precision medicine in real-world clinical practice. Eur. J. Paediatr. Neurol..

[28] Lee J., Lee C., Ki C.S., Lee J. (2020). Determining the best candidates for next-generation sequencing-based gene panel for evaluation of early-onset epilepsy. Mol. Genet. Genom. Med..

[29] Jang S.S., Kim S.Y., Kim H., Hwang H., Chae J.H., Kim K.J., Kim J.I., Lim B.C. (2019). Diagnostic Yield of Epilepsy Panel Testing in Patients With Seizure Onset Within the First Year of Life. Front. Neurol..

[30] Symonds J.D., Zuberi S.M., Stewart K., McLellan A., O’Regan M., MacLeod S., Jollands A., Joss S., Kirkpatrick M., Brunklaus A. (2019). Incidence and phenotypes of childhood- onset genetic epilepsies: A prospective population-based national cohort. Brain.

[31] Wilmshurst J.M., Gaillard W.D., Vinayan K.P., Tsuchida T.N., Plouin P., Van Bogaert P., Carrizosa J., Elia M., Craiu D., Jovic N.J. (2015). Summary of recommendations for the management of infantile seizures: Task Force Report for the ILAE Commission of Pediatrics. Epilepsia.

[32] Mastrangelo M., Galosi S., Cesario S., Renzi A., Campea L., Leuzzi V. (2022). Presenting Patterns of Genetically Determined Developmental Encephalopathies With Epilepsy and Movement Disorders: A Single Tertiary Center Retrospective Cohort Study. Front. Neurol..

[33] Ebrahimi-Fakhari D. (2018). Congenital Disorders of Autophagy: What a Pediatric Neurologist Should Know. Neuropediatrics.

[34] Girard J.M., Turnbull J., Ramachandran N., Minassian B.A. (2013). Progressive myoclonus epilepsy. Handb. Clin. Neurol..

[35] Ikeda A., Kumaki T., Tsuyusaki Y., Tsuji M., Enomoto Y., Fujita A., Saitsu H., Matsumoto N., Kurosawa K., Goto T. (2023). Genetic and clinical features of pediatric-onset hereditary spastic paraplegia: A single-center study in Japan. Front. Neurol..

[36] D’Gama A.M., Mulhern S., Sheidley B.R., Boodhoo F., Buts S., Chandler N.J., Cobb J., Curtis M., Higginbotham E.J., Holland J. (2023). Evaluation of the feasibility, diagnostic yield, and clinical utility of rapid genome sequencing in infantile epilepsy (Gene-STEPS): An international, multicentre, pilot cohort study. Lancet Neurol..

[37] Ho N.T., Kroner B., Grinspan Z., Fureman B., Farrell K., Zhang J., Buelow J., Hesdorffer D.C., Rare Epilepsy Network Steering Committee (2018). Comorbidities of Rare Epilepsies: Results from the Rare Epilepsy Network. J. Pediatr..

[38] Morrison-Levy N., Borlot F., Jain P., Whitney R. (2021). Early-Onset Developmental and Epileptic Encephalopathies of Infancy: An Overview of the Genetic Basis and Clinical Features. Pediatr. Neurol..

[39] Battaglia A., Hoyme H.E., Dallapiccola B., Zackai E., Hudgins L., McDonald-McGinn D., Bahi-Buisson N., Romano C., Williams C.A., Brailey L.L. (2008). Further delineation of deletion 1p36 syndrome in 60 patients: A recognizable phenotype and common cause of developmental delay and mental retardation. Pediatrics.

[40] Kolc K.L., Sadleir L.G., Scheffer I.E., Ivancevic A., Roberts R., Pham D.H., Gecz J. (2019). A systematic review and meta-analysis of 271 PCDH19-variant individuals identifies psychiatric comorbidities, and association of seizure onset and disease severity. Mol. Psychiatry.

[41] Trivisano M., Striano P., Sartorelli J., Giordano L., Traverso M., Accorsi P., Cappelletti S., Claps D.J., Vigevano F., Zara F. (2015). CHD2 mutations are a rare cause of generalized epilepsy with myoclonic-atonic seizures. Epilepsy Behav..

[42] Mullegama S.V., Mendoza-Londono R., Elsea S.H., Adam M.P., Feldman J., Mirzaa G.M., Pagon R.A., Wallace S.E., Bean L.J.H., Gripp K.W., Amemiya A. (1993–2023). MBD5 Haploinsufficiency. 27 October 2016 [updated 28 April 2022]. GeneReviews® [Internet].

[43] Emerson E. (2003). Prevalence of psychiatric disorders in children and adolescents with and without intellectual disability. J. Intellect. Disabil. Res..

[44] Helmstaedter C., Aldenkamp A.P., Baker G.A., Mazarati A., Ryvlin P., Sankar R. (2014). Disentangling the relationship between epilepsy and its behavioral comorbidities- the need for prospective studies in new-onset epilepsies. Epilepsy Behav..

[45] Maguire J., Jones N.C., Kanner A.M. (2021). Mechanisms of Psychiatric Comorbidities in Epilepsy. Psychiatric and Behavioral Aspects of Epilepsy. Current Perspectives and Mechanisms.

[46] Revdal E., Kolstad B.P., Winsvold B.S., Selmer K.K., Morken G., Brodtkorb E. (2023). Psychiatric comorbidity in relation to clinical characteristics of epilepsy: A retrospective observational study. Seizure.

[47] Symonds J.D., McTague A. (2020). Epilepsy and developmental disorders: Next generation sequencing in the clinic. Eur. J. Paediatr. Neurol..

[48] Guerrini R., Conti V., Mantegazza M., Balestrini S., Galanopoulou A.S., Benfenati F. (2023). Developmental and epileptic encephalopathies: From genetic heterogeneity to phenotypic continuum. Physiol. Rev..

[49] Knowles J.K., Helbig I., Metcalf C.S., Lubbers L.S., Isom L.L., Demarest S., Goldberg E.M., George A.L., Lerche H., Weckhuysen S. (2022). Precision medicine for genetic epilepsy on the horizon: Recent advances, present challenges, and suggestions for continued progress. Epilepsia.

[50] Oyrer J., Maljevic S., Scheffer I.E., Berkovic S.F., Petrou S., Reid C.A. (2018). Ion Channels in Genetic Epilepsy: From Genes and Mechanisms to Disease-Targeted Therapies. Pharmacol. Rev..

[51] Perucca P., Bahlo M., Berkovic S.F. (2020). The Genetics of Epilepsy. Annu. Rev. Genom. Hum. Genet..

[52] Coppola A., Cellini E., Stamberger H., Saarentaus E., Cetica V., Lal D., Djémié T., Bartnik-Glaska M., Ceulemans B., Helen Cross J. (2019). Diagnostic implications of genetic copy number variation in epilepsy plus. Epilepsia.

[53] Heilstedt H.A., Ballif B.C., Howard L.A., Kashork C.D., Shaffer L.G. (2003). Population data suggest that deletions of 1p36 are a relatively common chromosome abnormality. Clin. Genet..

[54] Torres F., Barbosa M., Maciel P. (2015). Recurrent copy number variations as risk factors for neurodevelopmental disorders: Critical overview and analysis of clinical implications. J. Med. Genet..

[55] Burk K.C., Kaneko M., Quindipan C., Vu M.H., Cepin M.F., Santoro J.D., Van Hirtum-Das M., Holder D., Raca G. (2023). Diagnostic Yield of Epilepsy-Genes Sequencing and Chromosomal Microarray in Pediatric Epilepsy. Pediatr. Neurol..

[56] Steenhof M., Kibæk M., Larsen M.J., Christensen M., Lund A.M., Brusgaard K., Hertz J.M. (2018). Compound heterozygous mutations in two different domains of ALDH18A1 do not affect the amino acid levels in a patient with hereditary spastic paraplegia. Neurogenetics.

[57] Ding J., Li X., Tian H., Wang L., Guo B., Wang Y., Li W., Wang F., Sun T. (2021). *SCN1A* Mutation-Beyond Dravet Syndrome: A Systematic Review and Narrative Synthesis. Front. Neurol..

[58] Scheffer I.E., Nabbout R. (2019). SCN1A-related phenotypes: Epilepsy and beyond. Epilepsia.

[59] Myers K.A., Burgess R., Afawi Z., Damiano J.A., Berkovic S.F., Hildebrand M.S., Scheffer I.E. (2017). De novo SCN1A pathogenic variants in the GEFS+ spectrum: Not always a familial syndrome. Epilepsia.

[60] Brunklaus A., Brünger T., Feng T., Fons C., Lehikoinen A., Panagiotakaki E., Vintan M.A., Symonds J., Andrew J., Arzimanoglou A. (2022). The gain of function SCN1A disorder spectrum: Novel epilepsy phenotypes and therapeutic implications. Brain.

[61] Fang Z., Hu C., Zhou S., Yu L. (2023). PIGW-related glycosylphosphatidylinositol deficiency: A case report and literature review. Neurol. Sci..

[62] Dontaine P., Kottos E., Dassonville M., Balasel O., Catros V., Soblet J., Perlot P., Vilain C. (2021). Digestive involvement in a severe form of Snyder-Robinson syndrome: Possible expansion of the phenotype. Eur. J. Med. Genet..

[63] Balestrini S., Mei D., Sisodiya S.M., Guerrini R. (2023). Steps to Improve Precision Medicine in Epilepsy. Mol. Diagn. Ther..

[64] Byrne S., Enright N., Delanty N. (2021). Precision therapy in the genetic epilepsies of childhood. Dev. Med. Child. Neurol..

[65] Demarest S.T., Brooks-Kayal A. (2018). From molecules to medicines: The dawn of targeted therapies for genetic epilepsies. Nat. Rev. Neurol..

[66] Helbig I., Ellis C.A. (2020). Personalized medicine in genetic epilepsies—Possibilities, challenges, and new frontiers. Neuropharmacology.

[67] Berg A.T., Langfitt J.T., Testa F.M., Levy S.R., DiMario F., Westerveld M., Kulas J. (2008). Global cognitive function in children with epilepsy: A community-based study. Epilepsia.

[68] Specchio N., Wirrell E.C., Scheffer I.E., Nabbout R., Riney K., Samia P., Guerreiro M., Gwer S., Zuberi S.M., Wilmshurst J.M. (2022). International League Against Epilepsy classification and definition of epilepsy syndromes with onset in childhood: Position paper by the ILAE Task Force on Nosology and Definitions. Epilepsia.

[69] Richards S., Aziz N., Bale S., Bick D., Das S., Gastier-Foster J., Grody W.W., Hegde M., Lyon E., Spector E. (2015). Standards and guidelines for the interpretation of sequence variants: A joint consensus recommendation of the American College of Medical Genetics and Genomics and the Association for Molecular Pathology. Genet. Med..

[70] Mu W., Lu H.M., Chen J., Li S., Elliott A.M. (2016). Sanger Confirmation Is Required to Achieve Optimal Sensitivity and Specificity in Next-Generation Sequencing Panel Testing. J. Mol. Diagn..

[71] Sands T.T., Choi H. (2017). Genetic Testing in Pediatric Epilepsy. Curr. Neurol. Neurosci. Rep..

[72] Wang J., Lin Z.J., Liu L., Xu H.Q., Shi Y.W., Yi Y.H., He N., Liao W.P. (2017). Epilepsy-associated genes. Seizure.

[73] Guerrini R., Balestrini S., Wirrell E.C., Walker M.C. (2021). Monogenic Epilepsies: Disease Mechanisms, Clinical Phenotypes, and Targeted Therapies. Neurology.

[74] Griffiths R., Huntley M. (1996). Griffiths Mental Development Scales-Revised: Birth to 2 Years (GMDS 0–2).

[75] Wechsler D. (2002). Wechsler Preschool and Primary Scale of Intelligence.

[76] Wechsler D. (2003). Wechsler Intelligence Scale for Children.

[77] Wechsler D. (1991). Weschler Intelligence Scale for Children.

[78] Rydz D., Shevell M.I., Majnemer A., Oskoui M. (2005). Developmental screening. J. Child. Neurol..

[79] American Psychiatric Association (2013). Intellectual disabilities. Diagnostic and Statistical Manual of Mental Disorders.

[80] Yang L., You C., Qiu S., Yang X., Li Y., Liu F., Zhang D., Niu Y., Xu L., Xu N. (2020). Novel and de novo point and large microdeletion mutation in *PRRT2*-related epilepsy. Brain Behav..

[81] Vlaskamp D.R.M., Callenbach P.M.C., Rump P., Giannini L.A.A., Brilstra E.H., Dijkhuizen T., Vos Y.J., van der Kevie-Kersemaekers A.F., Knijnenburg J., de Leeuw N. (2019). *PRRT2*-related phenotypes in patients with a 16p11.2 deletion. Eur. J. Med. Genet..

